# Performance of Fetal Medicine Foundation algorithm for first trimester preeclampsia screening in an indigenous south Asian population

**DOI:** 10.1186/s12884-021-04283-6

**Published:** 2021-12-04

**Authors:** Smriti Prasad, Daljit Singh Sahota, P. Vanamail, Akshatha Sharma, Saloni Arora, Anita Kaul

**Affiliations:** 1 Indraprastha Apollo Hospitals, New Delhi, India; 2 Department of Obstetrics and Gynaecology, The Chinese University of Hong Kong Prince of Wales Hospital, Shatin, Hong Kong SAR; 3 All India Institute of Medical Sciences, New Delhi, India; 4 New Delhi, India; 5 Head of the Department and Clinical Coordinator, Apollo Centre for Fetal Medicine, Indraprastha Apollo Hospital, New Delhi, India

**Keywords:** Pre-eclampsia, South Asian, Screening, 1st trimester, Uterine artery Pulsatility index, Mean arterial pressure, Placental growth factor, Pregnancy associated plasma protein-a

## Abstract

**Background:**

To evaluate the performance of the Fetal Medicine Foundation (FMF) preterm preeclampsia (PE) screening algorithm in an indigenous South Asian population.

**Methods:**

This was a prospective observational cohort study conducted in a tertiary maternal fetal unit in Delhi, India over 2 years. The study population comprised of 1863 women carrying a singleton pregnancy and of South Asian ethnicity who were screened for preterm pre-eclampsia (PE) between 11 and 14 weeks of gestation using Mean Arterial Pressure (MAP), transvaginal Mean Uterine Artery Pulsatility Index (UtAPI) and biochemical markers - Pregnancy Associated Plasma Protein-A (PAPP-A) and Placental Growth Factor.. Absolutemeasurements of noted biomarkers were converted to multiples of the expected gestational median (MoMS) which were then used to estimate risk for preterm PE < 37 weeks using Astraia software. Women with preterm PE risk of ≥1:100 was classified as as high risk. Detection rates (DR) at 10% false positive rate were calculated after adjusting for prophylactic aspirin use (either 75 or 150 mg).

**Results:**

The incidence of PE and preterm PE were 3.17% (59/1863) and 1.34% (25/1863) respectively. PAPP-A and PlGF MoM distribution medians were 0.86 and 0.87 MoM and significantly deviated from 1 MoM. 431 (23.1%) women had a risk of ≥1:100, 75 (17.8%) of who received aspirin. Unadjusted DR using ≥1:100 threshold was 76%.Estimated DRs for a fixed 10% FPR ranged from 52.5 to 80% depending on biomarker combination after recentering MoMs and adjusting for aspirin use.

**Conclusion:**

The FMF algorithm whilst performing satisfactorily could still be further improved to ensure that biophysical and biochemical markers are correctly adjusted for indigenous South Asian women.

## Background

Globally, preeclampsia (PE) remains an important contributor to maternal and perinatal morbidity and mortality with an incidence of 3-8% [1, 2] The ASPRE trial has demonstrated that women receiving prophylactic aspirin after a high risk screening test for preterm PE had a 62% reduction in the incidence of preterm PE [3].

The impetus is now on early identification of such high risk women by screening, with the model proposed by the Fetal Medicine Foundation (FMF), United Kingdom being the most widely used [4, 5]. FMF model based on maternal characteristics, medical history, biophysical parameters and biochemical markers has been able to achieve a detection rate of 75 and 47% at for a fixed false positive rate of 10% for PE < 37 and PE ≥ 37 weeks respectively [6].

The FMF algorithm was developed and initially tested in UK tertiary hospitals [4]. Whilst single center or pan Asian validation studies have been reported from Brazil, Belgium, Australia and Hong Kong; none however had a sizeable indigenous South Asian population [7–10]. The recently published pan-Asian study reported lower DRs compared to that achieved in the ASPRE study, with the authors acknowledging that their study findings were only applicable to East Asians [10].

The objective of the current study was to evaluate the performance of FMF algorithm for screening of PE in an indigenous South Asian population.

## Materials and methods

This was a prospective cohort study conducted at a tertiary maternal fetal unit in New Delhi, India between June 2017 and June 2019. South Asian women with a singleton pregnancy attending for their 1st trimester assessment of Downs syndrome risk were invited to participate in the study. Women were provided with pretest counselling and an information leaflet which described the PE screening test. Women gave written consent to participate in the study. Women were excluded, if at the time of ultrasound examination they were found to have major fetal abnormalities or an undiagnosed miscarriage.

Women completed a standard questionnaire to record physical size and sociodemographic characteristics, past and current medical and obstetric history as well as their current pregnancy details. Women underwent an ultrasound scan by one of six FMF certified sonographers, bilateral arm blood pressure measurement and blood draw procedure for measurement of Pregnancy Associated Plasma Protein-A (PAPPA) and placental growth factor (PlGF) concentrations on the Cobas e411 [Roche Diagnostics Ltd., Penzberg, Germany] immuno-analyser. All obtained blood samples were kept refrigerated at 4-8 °C pending analysis after initially being left to clot for 30 min.

Mean Arterial Pressure (MAP) was determined according to the standard FMF protocol using an Omron HEM-907 automated blood pressure device [11, 12]. All automated BP devices were regularly calibrated throughout the study.

Transvaginal scan was used to determine the left and right uterine artery PI (UTAPI) at the level of the internal os using a 2 cm sampling gate, an insonation angle < 30 degrees and only when the peak systolic velocity was ≥60 cm/sec. The mean UTAPI was calculated. Gestational age was determined using the fetal crown to rump length.

Women’s estimated risk for PE at the time of screening were determined using the FMF risk model incorporated in ASTRAIA software versions 2.4 to 3.4 [Astraia Gmbh, Germany]. Biomarkers were transformed to multiples of the expected median (MoM) and adjusted for maternal characteristics, smoking, mode of conception, South Asian ethnicity and immuno-analyser assay by the ASTRAIA software. A priori risk and adjusted risk for preterm PE in the ASTRAIA software were based on the competing risk model reported by Wright et al. and Akolekar et al [4, 13]. Women were designated as being at increased risk for preterm PE if their adjusted preterm PE risk was 1 in 100 or higher. Women having an adjusted risk for preterm PE of 1:100 or higher after July 2018 were counselled for the use of daily prophylactic aspirin (75/150 mg/day) after the ASPRE study findings were published and as per updated unit protocol.

Pregnancy outcomes were obtained from the hospital records or via telephone interview and documented in a secured electronic database. Women interviewed by phone were specifically asked about their antenatal BP measurement history as well as other symptoms related to development of PE. The re-analysis using de-identified data was exempted from review by the Institutional Ethical committee of Apollo Hospitals.

Hypertension in pregnancy was defined by the standard criteria of systolic blood pressure of ≥140 mmHg and/or a diastolic blood pressure of ≥90 mmHg on two occasions within a 4–6 h interval. PE was diagnosed as the appearance of hypertension and proteinuria (≥300 mg of protein in 24 h urine or protein creatinine ratio ≥ 30 mg/mmol, or 2+ on the dipstick) after 20 weeks of gestation [14].

### Sample size

The FMF 1st trimester screening model for preterm PE has a reported area under curve (AUC) of ≈ 0.9. We estimated that 26 preterm PE cases are needed to determine if the AUC of the test in our population was 0.9 assuming a standard error of the test is 5% for a type 1 error of 5% and power of 80%. Internal audit between 2006 and 2015 performed at our center indicated that the incidence of preterm PE was 3%. We therefore estimated that a minimum of 1891 women would need to be screened.

### Statistical analysis

One sample t-test was used to assess whether the log_10_ MAP, UtAPI, PAPP-A and PlGF MoMs distributions reported using the ASTRAIA software in non-PE pregnancies were significantly deviated from an expected mean of zero. Analysis of variance was performed to assess differences between log_10_ transformed MAP, UtAPI, PAPP-A and PlGF MoMs between pregnancies with no PE, preterm PE and term PE. Bonferroni correction was used to adjust for multiple comparisons. Analysis was performed using SPSS 22.0 (IBM Corp, NY, USA) with a *p* < 0.05 considered as statistically significant except where Bonferroni was used.

Preterm risks for PE using history combined with different biomarker combinations were determined by custom built software using the same published models used in our ASTRAIA software [13]. Individual biomarkers were re-centered prior to estimating risks if the one sample t-test indicated that mean of individual biomarker log_10_ MoM distributions in non-PE pregnancies were significantly deviated from the expected mean of zero using ASTRAIA software.

Receiver operating characteristic (ROC) curves were constructed and AUC, detection rates (DR) at a fixed FPR for screening for preterm PE were determined for differing biomarker combination. Screening performance was estimated after adjusting for prophylactic aspirin based on the previously reported assumptions that aspirin reduces the incidence of preterm PE by pre-specified probability of 0.6, confirmed in the ASPRE trial [3, 15] The number of women screened high risk taking aspirin who would have developed preterm PE had they not taken aspirin was estimated as 2.5 [1/(1-0.6)] times the number of observed cases of preterm PE in those taking aspirin. Reduction in preterm PE incidence due to aspirin was assumed to be independent of dose and compliance.

## Results

1975 women opted for screening for preterm PE over the 2 year period, of whom 60 (3%) were lost to follow-up. 52 (2.63%) of the screened pregnancies with known outcome were excluded from the analysis as either the pregnancy culminated in a spontaneous miscarriage or women sought a termination for reasons unrelated to PE.

The sociodemographic and pregnancy details, pregnancy outcome and biomarker distributions in the 1863 remaining pregnancies are presented in Table 1. Women who developed PE were significantly more likely to have a higher BMI with higher incidence of chronic hypertension and diabetes mellitus and a history of preeclampsia in their previous pregnancy. None of the women self-reported as being active smokers. Fifty-nine (3.2%) women developed PE of whom 25 (1.3%) had preterm PE.

**Table 1:** Socio-demographics and past and current obstetric history according to whether women did or did not develop pre-eclampsia (PE). Data are presented as mean ± Standard deviation (SD) or as number (%)

Characteristic	No Pre-eclampsia (*n* = 1804)	Pre-eclampsia (*n* = 59)	*p*-value
Age (years)	30.89 ± 4.04	31.40 ± 3.63	0.33
Weight (kg)	63.02 ± 10.85	65.47 ± 12.21	0.09
Height (cm)	159.57 ± 5.54	157.57 ± 5.07	0.006
Body Mass Index (kg/m^2^)	24.70 ± 4.18	26.35 ± 4.63	0.003
Crown Rump Length (mm)	63.61 ± 8.02	62.92 ± 6.22	0.513
Gestational at screening (days)	88.25 ± 4.43	88.12 ± 3.62	0.815
Assisted Conception	95 (5.3%)	2 (3.4%)	0.52
Chronic Hypertension	17 (0.9%)	13 (22.0%)	< 0.001
SLE/APS	6 (0.3%)	0 (0.0%)	0.66
Diabetes Mellitus	47 (2.6%)	6 (10.2%)	0.001
Past Obstetric History			
Nulliparous	1087 (60.3%)	35 (59.3%)	< 0.001
Parous no previous PE	692 (38.4%)	18 (30.5%)	
Parous with previous PE	25 (1.4%)	6 (10.2%)	
Screening Biomarkers			
log_10_ MAP MoM	−0.0029 ± 0.0307	0.0247 ± 0.0405	< 0.001
log_10_ UtAPI MoM	− 0.0146 ± 0.1325	−0.0105 ± 0.1440	0.154
log_10_ PAPP-A MoM	−0.0706 ± 0.2550	−0.1520 ± 0.2577	0.016
log_10_ PlGF MoM	−0.0608 ± 0.2153	−0.1701 ± 0.2305	0.001
Gestational at Delivery (days)	263.43 ± 30.16	254.83 ± 18.38	0.001
Male	963 (53.4%)	32 (54.2%)	0.897
Birth weight (g)	2933 ± 485	2465 ± 809	< 0.001

**Abbreviations:**; SLE- Systematic Lupus Erythematosus; Anti Phospholipid Antibody syndrome; MoM – Multiple of Median; MAP- Mean Arterial Pressure; UtAPI – Uterine Artery Pulsatility Index; PAPP-A – Pregnancy Associated Plasma Protein-A; PlGF – Placental Growth Factor

Four hundred and thirty one (23.1%) woman had a preterm PE risk of 1:100 or higher, 75 (17.4%) of whom took either 75 mg (*n* = 20) or 150 mg (*n* = 55) daily aspirin up to 36 weeks of gestation. No women screened low risk received aspirin. Nineteen of 34 women with term PE and 19 of 25 women with preterm PE risk had a preterm PE risk of 1:100 or higher. Ten of the 75 women taking aspirin developed PE, 6 had preterm PE and 4 term PE.

The log_10_ MAP, UtAPI, serum PAPP-A and PlGF MoM measurement distributions reported using the ASTRAIA software in unaffected pregnancies were Gaussian but with their distribution means statistically significantly deviated from an expected mean of zero (*p* < 0.001 for all). However, the extent of the deviation of MAP and UtAPI MoMs was not considered clinically significant as the median of their respective MoM distributions was 0.99MoM. In contrast, the median of the PAPP-A and PlGF MoMs distributions were 0.86 and 0.87 MoM respectively. PAPP-A and PlGF MoMs distributions were therefore re-centered by dividing the original reported ASTRAIA MoM values by 0.86 and 0.87 respectively to give a corrected MoM with median values of 1 MoM. These new revised and corrected MoMs were used in subsequent analysis and assessment of screening performance using the different biomarker combinations. Retrospective recalculation of the preterm PE adjusted risk after correcting for 11-13% shift in the PlGF and PAPPA MoMs distributions reduced SPR by ≈2% from 23.1 to 21.6% using a 1:100 cut-off.

Assessment of preterm PE screening performance was therefore based on the assumption that 40 women in total would have had preterm PE, 6 who were screened low risk and 34 who were screened high risk constituted from 13 screened high risk who did not take aspirin, 6 screened high risk who took aspirin plus 15 whose preterm PE was prevented or delayed by aspirin [3, 15].

**Fig. 1:**
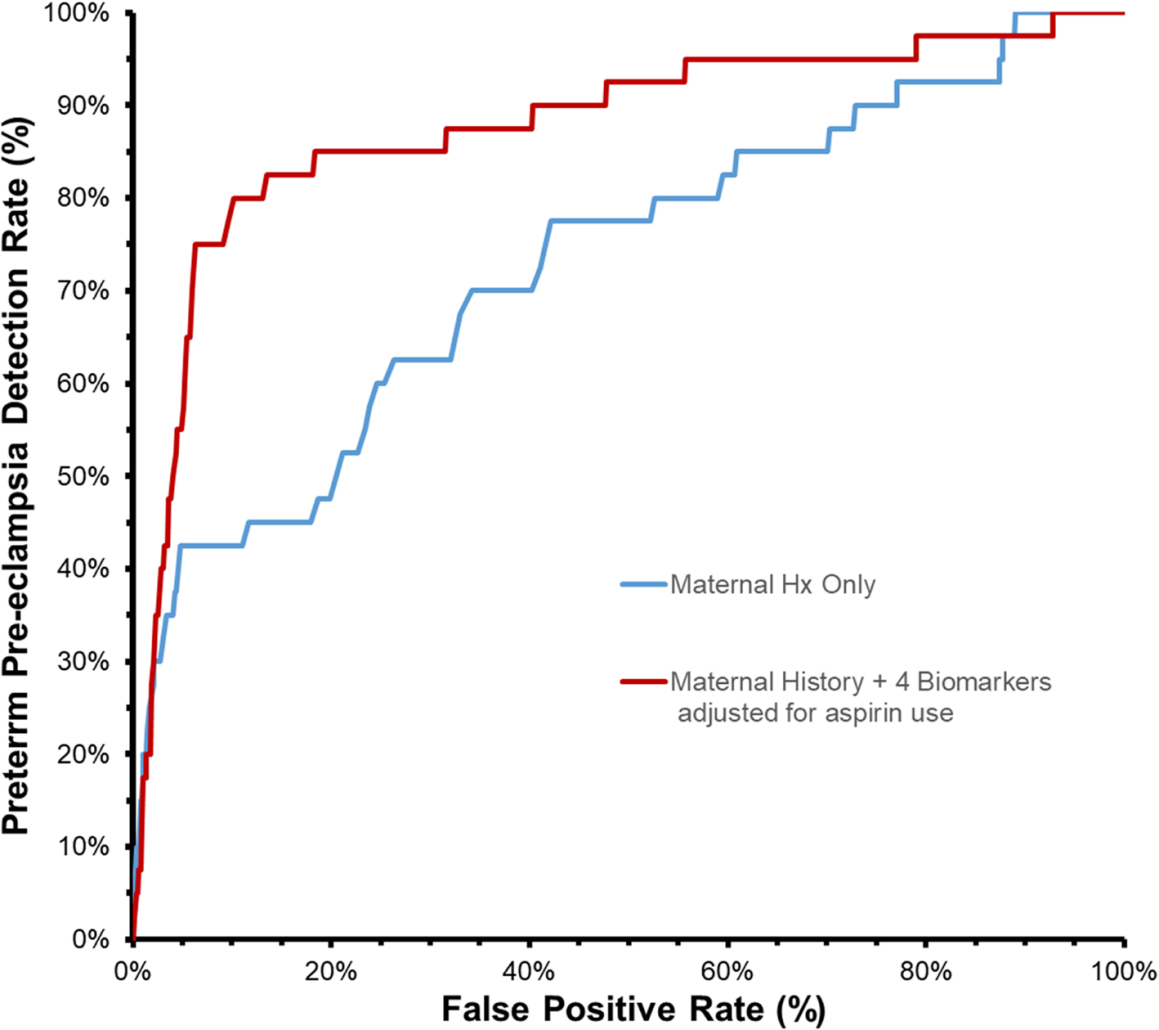
Receiver operational curves showing the screening performance for preterm pre-eclampsia using maternal history and maternal history plus the four biomarkers (MAP, UtAPI, PlGF, PAPP-A) after assuming that aspirin would reduce incidence of preterm pre-eclampsia by 60%.(3,15)

Fig. 1 shows the ROC curves for screening for preterm PE based on maternal history alone as well as 4 biomarkers in combination with maternal history after re-centering MoMs and adjusting for aspirin use. The respective AUC were 0.76 (95%CI 0.63-0.89) and 0.96 (95%CI 0.95-0.98). Table 2 summarizes the expected DR for different biomarker combinations at a fixed 10% FPR. The estimated DR for preterm pre-eclampsia for a 10% FPR using the FMF triple combination (MAP, UtAPI, PlGF) was 77.5% and increased to 80.0% using all 4 biomarkers (MAP, UtAPI, PlGF, PAPPA).

**Table 2:** Estimated detection rates (DR) for preterm pre-eclampsia at a false positive rate (FPR) of 10% in our indigenous South Asian population using maternal history in combination with Mean Arterial Pressure (MAP), Mean Uterine Artery Pulsatility Index (UtAPI), Placental Growth Factor (PlGF)

Preterm Pre-eclampsia Screening Test	Risk Cutoff	DR @ 10% FPR
History Alone	1:47	40.0%
+ MAP	1:53	52.5%
+ UtAPI	1:42	57.5%
+ PlGF	1:40	62.5%
+ MAP+ UtAPI + PlGF	1:49	77.5%
+ MAP + UtAPI + PlGF + PAPPA	1:44	80.0%

## Discussion

### Main findings

We undertook this study to assess the FMF PE screening algorithm available at the time the study was conducted in an indigenous South Asian population. We noted lower median PAPP-A and PlGF MoMs in our cohort as compared to that reported in ‘Whites/Caucasians’. This would bias PE screening performance towards higher SPR and higher DR. The observed SPR in our study was 23% using the risk available at time of screening and a 1:100 cut off. Had PAPPA and PlGF MoMs been correctly centered then SPR would have been 21.6% using the same 1:100 threshold. Our findings would suggest that screening for preterm PE using the 1st trimester multi-marker screening approach in India would be an improvement on the general practice of screening by maternal characteristics alone.

### Interpretations

At present FMF algorithm incorporated into risk calculation software is used on an as is basis with the assumption that population characteristics of women with regard to PE incidence rates and physical size and biomarker levels used to create the FMF models in the United Kingdom would be equally applicable elsewhere.

The lower Biochemical MoMs in our cohort is consistent with those recently reported in a pan Asian study by Chaemsaithong and colleagues [16]. This study reported that PlGF MoM using the FMF MoMing model in their study were 16% lower despite adjusting for ethnicity when the MoM was calculated [16]. In contrast to our data, the median PlGF MoM in the only Indian center, located in Southern India, in Chaemsaithong et als study was 20% higher than ‘Whites/Caucasians’ and approximately 40% higher than the median in our own cohort [16]. This is in contrast to the study by Tan et al. which reported that PlGF MoM levels in East and South Asians in their UK population were respectively 8 and 18% higher than ‘White/Caucasians’ women after adjusting for other factors [17]. Marked difference in body dimensions and adiposity indices have been noted between North and South Indians [18]. How these regional variations in anthropometry would affect PlGF MoMs remains unclear. Further studies would be needed to assess inter-regional variation in maternal biochemistry levels across different Indian populations using standardized blood collection and processing protocol to determine whether levels reported in the earlier study are increased due to delayed processing of the blood sample or due to potential differences between analysis of fresh and previously frozen serum samples.

The estimated DR for preterm PE screening after re-centering MoMs and adjusting for aspirin use for a 10% FPR using the 4 biomarker combination would be 80% similar to the 77% estimated by Akolekar et al. using the same risk model [4]. Both our DR and those reported for UK populations are significantly higher than the 64% reported in East Asian women [10]. An earlier study by Cheng et al. similarly reported lower DRs for early onset PE [19]. Had we used only MAP, UTAPI and PlGF, the same biomarkers combination used in both East Asian and ASPRE studies, [3, 10] the estimated DR after adjusting for aspirin would be 78% at a 10% FPR, again higher than East Asian studies but almost identical to the 76.6% reported in ASPRE [20]. This would suggest that the FMF screening model should perform equally as effectively in screening for PE provided the biochemistry is correctly adjusted.

### Strengths and limitations

The strengths of the study were its prospective design with high follow up rates (about 97%), that all biomarkers were assessed by FMF accredited sonographers using FMF described protocols except where stated and that we assessed screening performance after adjusting for aspirin use. Despite using an automated device to measure MAP and transvaginal rather than transabdominal route to assess UtAPI the MAP and UtAPI MoM distributions both had median close to expected 1 MoM. We used a transvaginal approach as it is an integral part of first trimester anatomy assessment in our unit and readily accepted by women attending our clinic. Whether trans-vaginal PI measurements are comparable, higher or lower than trans-abdominal PI remains unclear with some studies indicating trans-abdominal PI are higher than transvaginal whereas other studies indicate the opposite [21, 22]. When adjusting for aspirin treatment effect we assumed that it was independent of dosage and compliance, an assumption previously made by others [3, 10, 15]. Aspirin treatment effect has been shown to be dose dependent and that Afro-Caribbean and South Asian in the UK were less likely to comply [23, 24]. Further studies are therefore needed to assess South Asian women’s motivation for accepting or not accepting aspirin if screened high risk for prterm PE and their aspirin use compliance. Such studies would allow assessment of aspirin prophylaxis efficacy in preventing preterm PE as well as allowing permitting more accurate estimation of the preterm PE screening test sensitivity.

The low rate of cigarette smoking in pregnancy in our study is consistent with the Indian National Family Health Survey-3 survey, which reported that only 1% of pregnant women reported that they smoked as opposed to higher usage of smokeless forms of tobacco [25].

## Conclusion

The FMF screening algorithm for preterm PE performing satisfactorily could still be further improved to ensure that biomarkers are correctly adjusted for indigenous South Asian women. Further large scale studies are however needed in indigenous South Asian populations to confirm our biomarker findings, to assess cost-effectiveness of screening and confirm our findings that hazard rates for preterm PE estimated by the competing risk model incorporated in the FMF algorithm are applicable locally. The alternative would be to develop a localized model to derive estimates for the a priori risk and biomarker MoMs whilst still adopting the FMF concept of a competing risk approach.

## Data Availability

Available from corresponding author on request.
